# Characteristics of different types of *Helicobacter pylori*: New evidence from non-amplified white light endoscopy

**DOI:** 10.3389/fmicb.2022.999564

**Published:** 2023-01-13

**Authors:** Weidong Liu, Wenjie Kong, Wenjia Hui, Chun Wang, Qi Jiang, Hong Shi, Feng Gao

**Affiliations:** ^1^College of Life Science and Technology, Xinjiang University, Urumqi, China; ^2^Department of Gastroenterology, People's Hospital of Xinjiang Uygur Autonomous Region, Urumqi, China; ^3^Xinjiang Clinical Research Center for Digestive Diseases, Urumqi, China; ^4^Department of Pathology, People's Hospital of Xinjiang Uygur Autonomous Region, Urumqi, China; ^5^Department of Gastroenterology, ZhongShan Hospital, Fudan University, Shanghai, China

**Keywords:** endoscopy, *Helicobacter pylori*, CagA Ab, gastritis—microbiology, urea breath test

## Abstract

**Background:**

Different types of *Helicobacter pylori (H. pylori)* were analyzed to determine their infection characteristics using serology, pathology, and non-magnification white light endoscopy combined with the Kimura–Takemoto classification, and the regular arrangement of collecting venules (RAC) as well.

**Materials and methods:**

A retrospective analysis of 685 inpatients who have completed the ^14^C-urea breath test, the *H. pylori* antibody typing classification, the serum gastric function tests (PGI/PGII/G-17), the endoscope detection, and the pathological examinations.

**Results:**

The levels of PGI, PGII, and G-17 were in descending order from the type I *H. pylori* infection group to the type II *H. pylori* infection group than the control group (*F* = 14.31; 26.23; 9.12, *P* < 0.01). Using the Kimura–Takemoto classification, there were significant differences among the three groups of different degrees of atrophy ( $\chi^{2}$ =29.81; 482.78; 292.5, *P<* 0.01). Based on the characteristics of RAC, the *H. pylori* infection rates were in descending order from the type I *H. pylori* infection group to the type II *H. pylori* infection group than the control group ( $\chi^{2}\;$ = 200.39; 174.72; 143.51, *P* < 0.01). The type I *H. pylori* infection group had higher grades than those of the type II *H. pylori* infection group in the OLGA and OLGIM staging systems, while the differences are statistically significant only in the OLGA staging system ( $\chi^{2}$ =10.63, *P* < 0.05).

**Conclusion:**

With the aid of non-amplified white light endoscopy, we found new evidence of type I *H. pylori* infection accelerating the progression of gastric mucosal atrophy through the degree of atrophy and the range of infection, whereas type II *H. pylori* infection has a low ability of migration and atrophy progression. Individual virulence factor-based eradication therapy may be a better choice in future.

## 1. Introduction

The major sites of *Helicobacter pylori* (*H. pylori*) infection are the stomach and duodenal bulbs, which are significantly associated with chronic gastritis, gastric mucosa atrophy and erosion, peptic ulcer, MALT lymphoma, and gastric cancer (Smith et al., 2017). In 1994, *H. pylori* were classified as a class I biological carcinogen by the World Health Organization (Ferreira et al., 2014). In 2015, the *Kyoto Global Consensus Report on Helicobacter pylori* identified *H. pylori* as an infectious disease (Sugano et al., 2015). In 2022, the United States Department of Health and Human Services listed *H. pylori* as a definite carcinogen. *Helicobacter pylori* are a highly heterogeneous bacterium, from which many virulence factors have been isolated and identified. Cytotoxin-associated gene A (*CagA*) and Vacuolating cytotoxin gene A (*VacA*) have been extensively studied as the virulence markers of *H. pylori*, since carrying these two genes has made *H. pylori* closely associated with the occurrence and development of many gastric diseases (Chey et al., 2017; Lee et al., 2021). Recent studies have shown that the CagA of *H. pylori* can cause genomic instability induced by BRCNESS. Moreover, *H. pylori*’s CagA can cause gastric cancer through a “hit and run” mechanism in the absence of p53 (Imai et al., 2021).

Clinically, based on the antibody expression of CagA and VacA, *H. pylori* can be classified into type I *H. pylori* infection (CagA^+^/VacA^+^) and type II *H. pylori* infection (CagA^−^/VagA^−^). Previous studies have found that type I *H. pylori* infection may contribute to the progression of gastric mucosal atrophy through its higher virulence factors and migration ability; however, there is no direct evidence to support this in the real gastric condition (Liu et al., 2021; Zhang et al., 2022). In this study, different types of *H. pylori* were analyzed to determine their infection characteristics using serology, pathology, and non-magnification white light endoscopy combined with Kimura–Takemoto classification, and the regular arrangement of collecting venules (RAC) as well. We have explored characteristics of different types of *H. pylori* causing gastric mucosa atrophy through virulence factors and migration ability in a specific population (50–60 years old), which may provide a new theoretical basis for clinical individual eradication therapy.

## 2. Materials and methods

### 2.1. Subject investigated

A retrospective analysis of 745 inpatients in the Department of Gastroenterology of Xinjiang Urumqi People’s Hospital from March 2019 to January 2022, and 685 cases were eventually included given the exclusion criteria. All inpatients were Han Chinese, aged 50–60 years, and have completed the ^14^C-urea breath test, the *H. pylori* antibody typing classification, the serum gastric function tests (PGI/PGII/G-17), and the endoscope detection and the pathological examinations. The exclusion criteria included the following: previous history of gastric cancer and gastric cancer surgery; active bleeding and other serious systemic diseases; previous eradication of *H. pylori*; discordant results from the ^14^C-urea breath test and the *H. pylori* antibody typing classification; and incomplete endoscopic data. G*Power was used to calculate the sample size: select tests—Goodness-of-fit tests: Contingency tables, effect size = 0.3, α = 0.05, 1−β = 0.95. The results of pre-investigation were taken as parameters, and the minimum sample size required for calculation was 342 cases. The sample size of the study was 685 cases, which could ensure reliable results. The study was approved by the ethics committee of the People’s Hospital of Xinjiang Uygur Autonomous Region (KY2019051528).

### 2.2. ^14^C-urea breath test

The inpatients were required to take a ^14^C urea capsule on an empty stomach or 2 h after meals and sit for 25 min, and then blow into the gas collector for about 3 min until the liquid indicator color became colorless. Then, 4.5 ml scintillation solution to the gas collector was added and mixed upside down three times before being sent to the *H. pylori* detector (HUBT01) for 1 min. The sample was determined as positive for *H. pylori* infection if the detection value ≥100 dpm, and negative for the detection value <100 dpm.

### 2.3. *Helicobacter pylori* antibody typing classification

To type and classify *H. pylori* antibodies, 2–3 ml venous blood was collected from hospitalized patients, and then the serum was obtained by centrifugation at 3,500 rpm/10 min. The *H. pylori* antibody typing classification kit (immunoblotting method) was provided by Shenzhen Blot Biological Products, Shenzhen, China, Co., Ltd. The imprinting membrane strip was qualitatively compared with the standard strip after binding to serum antibodies, enzyme-linked reaction, color reaction, and termination of the reaction. Positive for type I *H. pylori* infection: either or both of the CagA and VacA zones appeared simultaneously. Positive for type II *H. pylori* infection: either or both of urease A (UreA) and UreB zones but no CagA or VacA zone were found. Negative results: no positive zone was found in the color zone.

### 2.4. Serum gastric function tests

For the serum gastric function test, serum was obtained by centrifugation of 3 ml venous blood at a rate of 3,500 rpm/10 min. The serum gastric function assay kit (Biohit, Hefei, China) was used to follow its instructions: 80 μl serum was added into the sample hole of the test card and kept for 15 min, then it was detected by fluorescence immunoassay (HIT-9A). The normal reference range of PGI was 70–165 μg/L, the normal reference range of PGII was 3–11 μg/L, the normal reference range of PGI/PGII was >7, and the normal reference range of G-17 was 1–7 pmol/L.

### 2.5. Endoscope detection

To assess the status and extent of *H. pylori* infection, an endoscopy was performed. All patients underwent endoscopy performed by an endoscopist with 5 years of standardized training, using either GIF-H260 or GIF-HQ290 from Olympus, Japan, and biopsies were performed according to the new Sydney standard. The range and degree of endoscopic atrophy were classified according to the Kimura–Takemoto classification, and the mucosal lesions were divided into 0, C1, C2, C3, O1, O2, and O3, and two senior endoscopists were responsible for determining the degree of atrophy (see Appendant.1). The status and range of *H. pylori* infection were evaluated in conjunction with the characteristics of endoscopic RAC (see Appendant.2).

### 2.6. Pathological examinations

Endoscopic biopsies were formalin overnight, paraffin-embedded the next day, sectioned with a sectioning machine, HE stained by an automated immunohistochemical machine, and biopsied by a pathologist based on the most common chronic gastritis; five histological changes (*H. pylori*, chronic inflammatory lesions, motility, atrophy, and intestinal metaplasia) were assessed and the degree of each histological change was assessed as nil, mild, moderate, and severe. The grading method was based on the *consensus of pathological diagnosis for biopsy of chronic gastritis and epithelial tumor of the gastric mucosa* (2017), combined with the updated Sydney System’s visual analog scales. The pathological diagnosis report included histological changes in biopsy specimens from each site. The OLGA and OLGIM staging systems were used to assess chronic gastritis with gastric atrophy. The OLGA staging system ranks the degree of pathological atrophy as stage 0, stage1, stage 2, stage 3, and stage 4 (see Appendant.3A); and the OLGIM staging system ranks the degree of pathological intestinal metaplasia as stage 0, stage1, stage 2, stage 3, and stage 4 (see Appendant.3B).

### 2.7. Statistical analysis

All data were analyzed by SPSS 19.0. The metering data were presented as $\bar{x}\;$ ± S, variance analysis or non-parametric tests were used for the mean value among groups, and the Mann–Whitney U-test was used for the comparison between the two groups. The counting data were represented by the number of cases and percentage. Pearson Chi-Square and Fisher’s exact test were used for comparison between groups. The difference was considered statistically significant at *P* < 0.05.

## 3. Results

### 3.1. Analyses of general data

The study included 745 Han Chinese inpatients aged 50–60 years, and 685 patients were finally enrolled given the exclusion criteria. There were 355 male and 330 female patients with an average age of 55.4 ± 3.3 years. According to the *H. pylori* antibody typing classification, these inpatients were divided into three groups. Among the 291 patients with type I *H. pylori* infection, 148 were male and 143 were female patients, and the average age was 55.3 ± 3.2 years. Among the 110 patients with type II *H. pylori* infection, there were 54 male and 56 female patients with an average age of 55.4 ± 3.3 years. The control group was made of 153 male and 131 female patients, 284 in total with an average age of 55.5 ± 3.3 years. There was no significant difference in sex and age between groups ( $x^{2}\;$ = 0.91, *p* = 0.63; $x^{2}\;$ = 0.45, *P* = 0.80, *P* all > 0.05).

### 3.2. Expression of serum gastric function tests levels in different types of *Helicobacter pylori*

**Figure 1:**
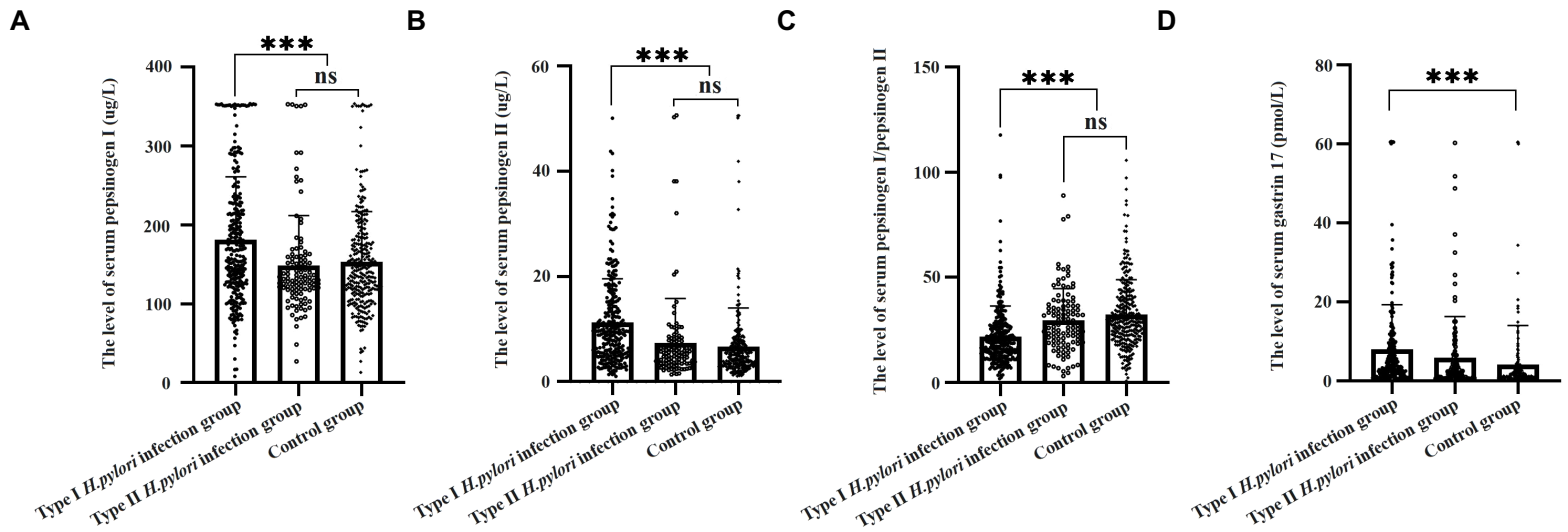
Serum gastric function of different types of *Helicobacter pylori* infection.

Serum gastric function tests revealed that expression levels of PGI were higher in the type I *H. pylori* infection group (181.0 ± 79.9 μg/L) than those in the type II *H. pylori* infection group (148.8 ± 62.9 μg/L) and the control group (153 ± 63.8 μg/L), and the differences were statistically significant (*F* = 14.1, *P* < 0.01), as shown in Figure 1A. The expression levels of PGII were higher in the type I *H. pylori* infection group (11.3 ± 8.3 μg/L) than those in the type II *H. pylori* infection group (7.4 ± 8.4 μg/L) and the control group (6.7 ± 7.4 μg/L), and the differences were statistically significant (*F* = 26.2, *P* < 0.01), as shown in Figure 1B. The expression levels of PGR were lower in the type I *H. pylori* infection group (22 ± 14.5) than those in the type II *H. pylori* infection group (29.5 ± 15.2) and the control group (32.3 ± 16.6), and the differences were statistically significant (*F* = 33.2, *P* < 0.01), as shown in Figure 1C. The expression levels of G-17 were higher in the type I *H. pylori* infection group (8 ± 11.4 pmol/L) than those in the type II *H. pylori* infection group (5.9 ± 10.4 pmol/L) and the control group (4.2 ± 9.8 pmol/L), and the differences were statistically significant only between the type I *H. pylori* infection group and the control group (*F* = 9.1, *P* < 0.01), as shown in Figure 1D.

### 3.3. Characteristic analysis of atrophy of different types of *Helicobacter pylori* infection under endoscopy

**Figure 2:**
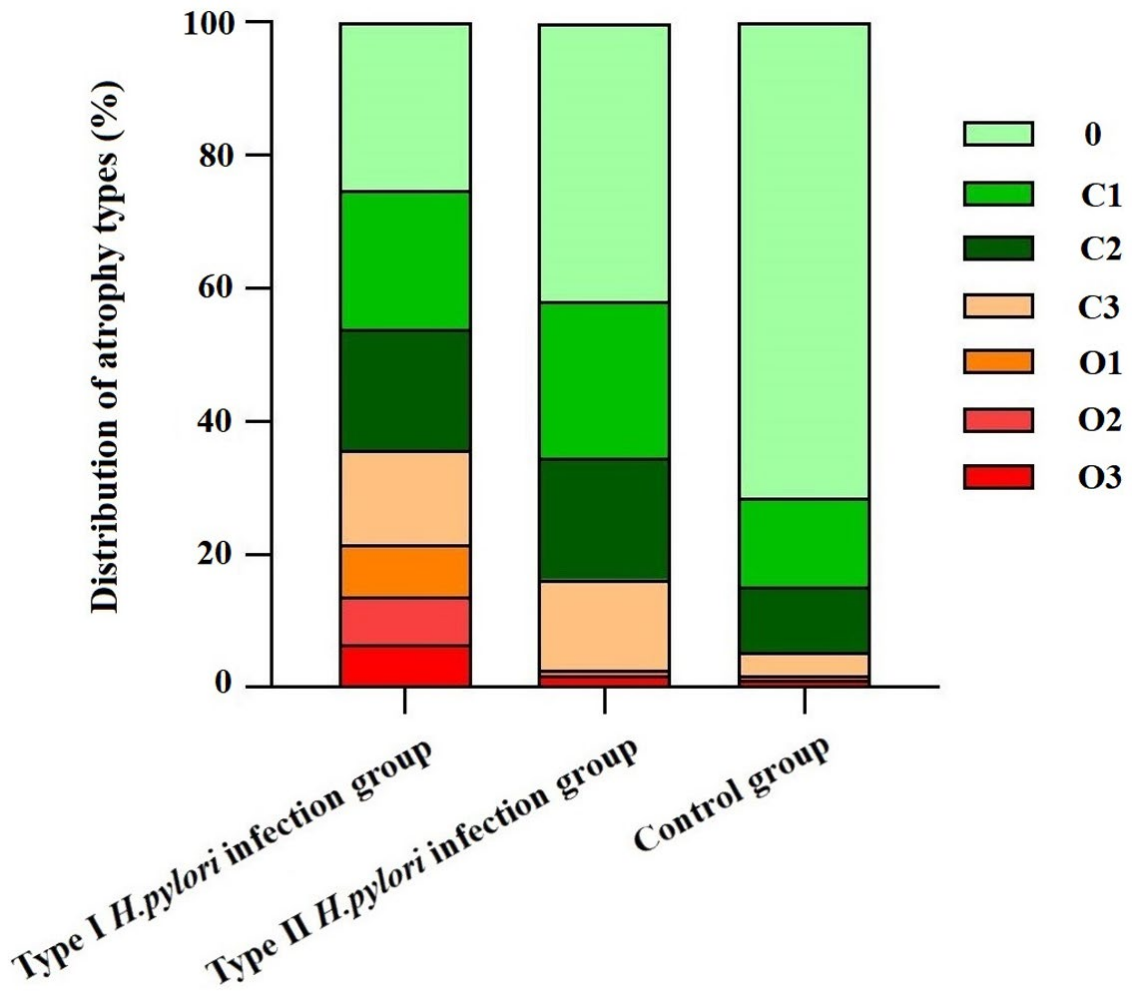
Distribution of different types of *Helicobacter pylori* infection in Kimura–Takemoto classification.

The composition ratio of different degrees of atrophy in the Kimura–Takemoto classification was statistically different among the type I *H. pylori* infection group, the type II *H. pylori* infection group, and the control group ( $x^{2}$ =29.81; 482.78; 292.5, *P <* 0.01). Intergroup comparison has shown that only the composition ratio of C1 had no significant difference between the type I *H. pylori* infection group and the type II *H. pylori* infection group ( $x^{2}$ =0.34, *P* > 0.05), as shown in Figure 2.

### 3.4. Distribution of different types of *Helicobacter pylori* infection under endoscopy

**Figure 3:**
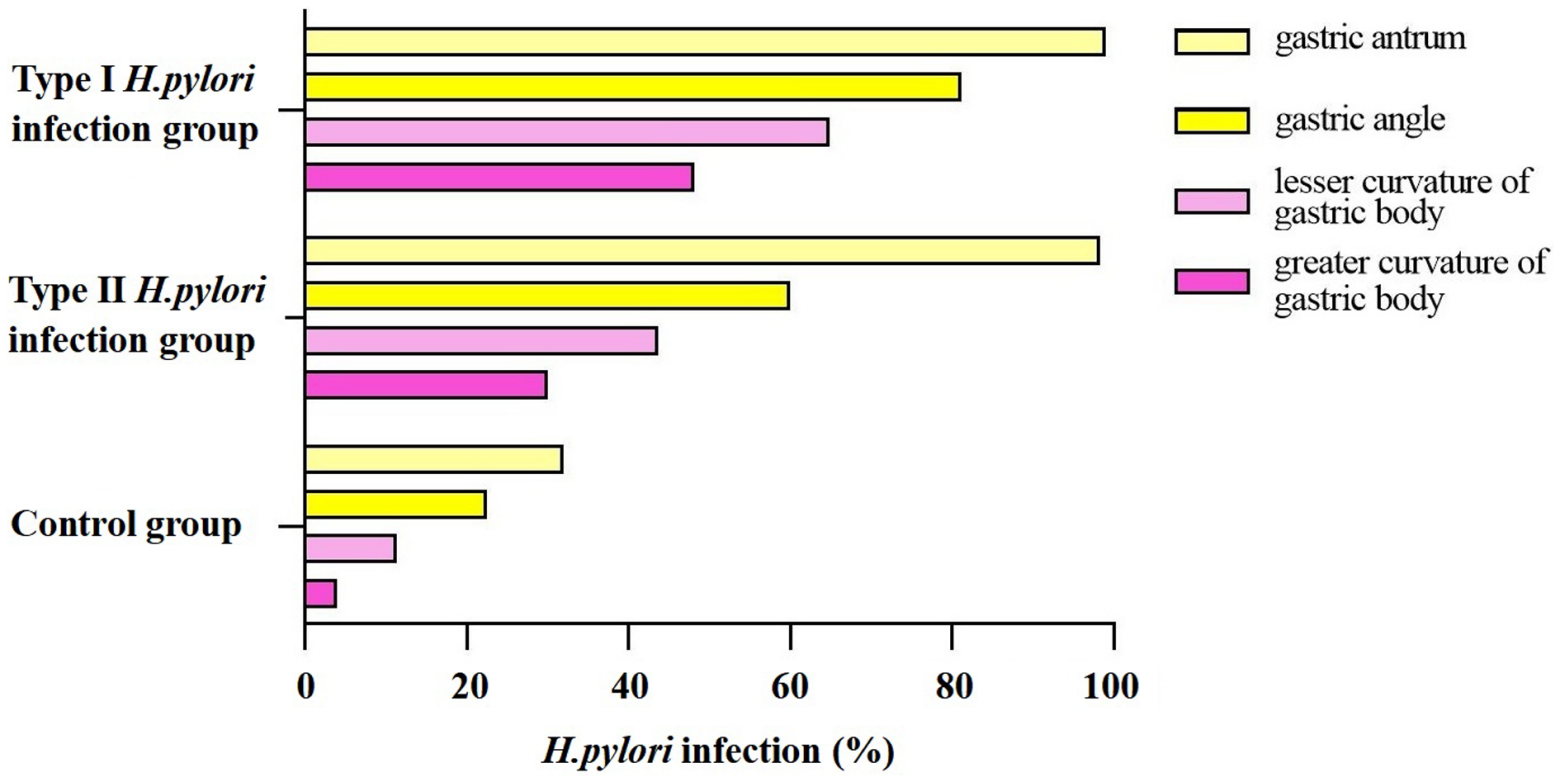
Distribution of different types of *Helicobacter pylori* infection in different parts of the stomach.

Regular arrangement of collecting venules is mainly distributed in the gastric angle and gastricum, and the site and range of *H. pylori* infection can be located and evaluated by the absence of RAC; therefore, the status of *H. pylori* infection is mainly estimated by the characteristics of RAC under endoscopy. The results showed that the type I *H. pylori* infection group and the type II *H. pylori* infection group expressed higher characteristics of infection in gastric antrum ( $x^{2}$ = 2, *P* > 0.05). According to the characteristics of RAC, the *H. pylori* infection rates in the angular lesser curvature of the stomach and the greater curvature of the stomach were in descending order from the type I *H. pylori* infection group to the type II *H. pylori* infection group than the control group, and the differences were statistically significant ( $x^{2}$ = 200.39; 174.72; 143.51, *P* < 0.01), as shown in Figure 3.

### 3.5. Different types of *Helicobacter pylori* infection in OLGA and OLGIM staging system

**Figure 4:**
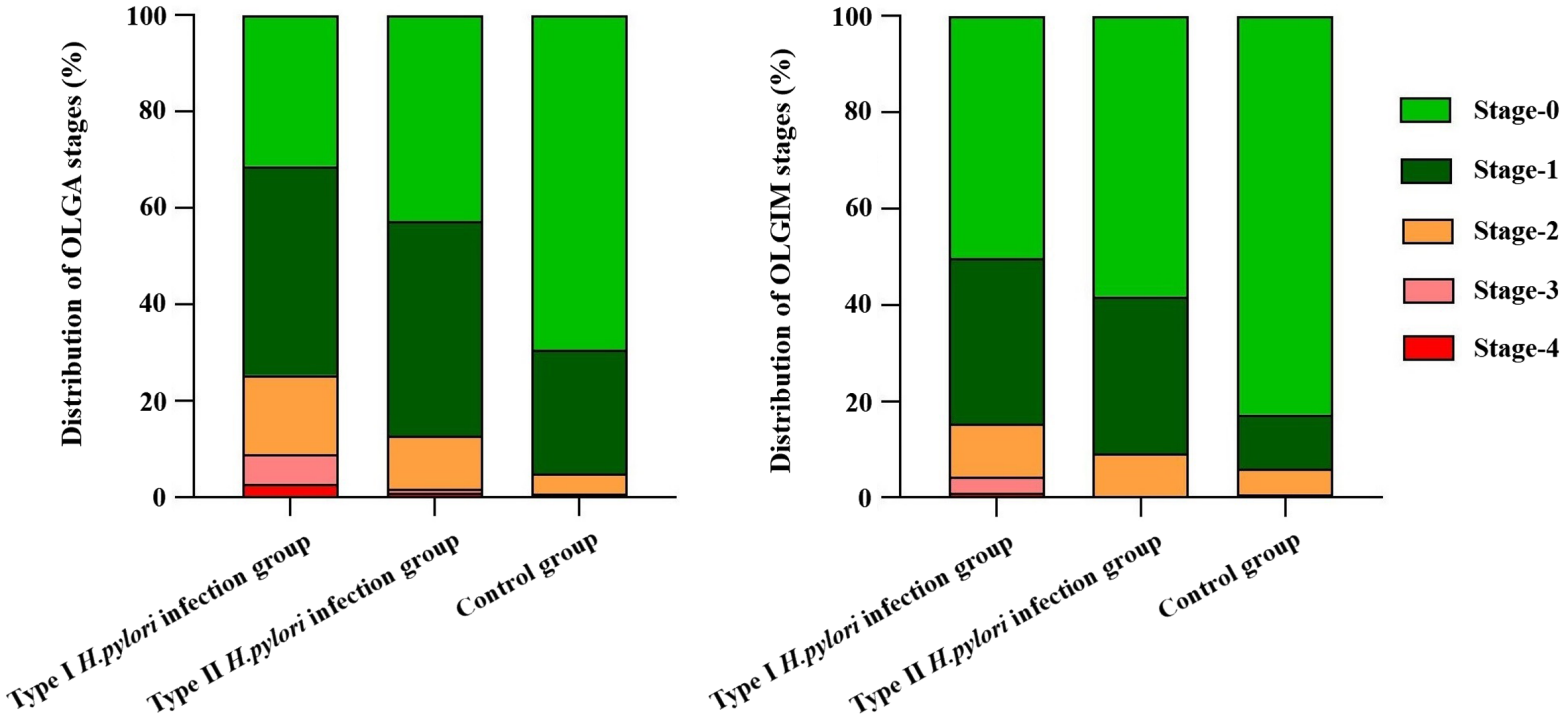
Different types of *Helicobacter pylori* infection in OLGA and OLGIM staging system.

In the OLGA and OLGIM staging systems, the composition ratio of the type I *H. pylori* infection group, the type II *H. pylori* infection group, and the control group is shown in Figure 4. Specifically, the type I *H. pylori* infection group graded higher in the OLGA and OLGIM staging systems than those in the control group, and the differences were statistically significant ( $x^{2}$ = 95.45; 70.23, *P* < 0.01). The type II *H. pylori* infection group graded higher in the OLGA and OLGIM staging systems than those in the control group, and the differences were statistically significant ( $x^{2}$ = 26.62; 30.05, *P* < 0.01). The type I *H. pylori* infection group graded higher in the OLGA and OLGIM staging systems than those in the type II *H. pylori* infection group, and the differences were statistically significant only in the OLGA staging system ( $x^{2}\;$ = 10.63, *P* < 0.05).

## 4. Discussion

*Helicobacter pylori* have a high intraspecific genetic diversity, and most studies have focused on the identification of strain specificity related to gastric cancer (Tissera et al., 2022). The pathogenic mechanism of *H. pylori* may be related to many pathogenic factors of the bacteria, such as genes encoding outer membrane proteins (babA, oipA, sabA, and hopQ), exercise genes (flaA and flaB), and iceA, of which the CagA, VacA, activating protein A of peptic ulcer, and adhesins are particularly important (Da Costa et al., 2015). The CagA may interact with several host proteins after being transmitted to the cytoplasm, by either EPIYA phosphorylation-dependent or non-dependent, to regulate key cellular functions such as proliferation, apoptosis, inflammation, and genome integrity (Knorr et al., 2019; Kontizas et al., 2020). At present, clinical studies of type I *H. pylori* infection are closely related to the severity of many diseases, such as atrophic gastritis, ulcers, gastritis cancer, arteriosclerosis, and myasthenia gravis (El Khadir et al., 2021; Li et al., 2021; Wu and Chen, 2021). Atrophic gastritis is led by *H. pylori* infection and has a high risk of gastric cancer (Kato et al., 2019). Although eradication of *H. pylori* may reduce the risk of gastric cancer in the general population, nevertheless, it does exist and is associated with the degree of atrophy and intestinal metaplasia (Shibagaki et al., 2021). Specific *H. pylori* antibodies and CagA may act together on the progression of intestinal metaplasia in non-atrophic gastritis (Song et al., 2022). A large cohort of the study found an increased risk of cascade in patients positive for *H. pylori*, and they progressed from chronic gastritis to atrophic gastritis, eventually leading to intestinal metaplasia (Ohata et al., 2004). It takes about 10 years for atrophic gastritis to progress to atrophy or intestinal metaplasia, but 11.6 years to atrophic gastritis and 11.4 years to intestinal metaplasia for 95% of those people in precancerous conditions (Kodama et al., 2012). At the age of 60 years, the gastric mucosa will undergo atrophy and intestinal metaplasia, and the eradication of *H. pylori* at the right time can slow down the atrophic gastritis process (Chey et al., 2017).

Pepsin is an inactive precursor of pepsin in gastric juice, which can be divided into PGI and PGII subpopulations according to their biochemical properties, immunogenicity, cell origin, and tissue distribution. The serum gastric function tests can be used as an index for gastric diseases (Chen et al., 2018; Yuan et al., 2020). The PGI is secreted by the principal cells of the gastricum and gastric fundus and the PGII is secreted by the pyloric gland, fundus gland, Brunner gland, and cardiac gland. The G-17 is secreted by the G cells of the antrum. Serum PGI, PGII, and G-17 levels are related to one’s living habits, environment, *H. pylori* infection, sex, age, smoking, as well as alcohol consumption (Sjomina et al., 2018). The PGI, PGII, and G-17 levels are positively correlated with the activity and degree of inflammation of chronic gastritis in the antrum and gastricum, while PGR was negatively correlated with inflammation (Li et al., 2022). Serum PGI, PGII, PGR, and G-17 may indirectly reflect the secretory site of gastric mucosal lesions. To eliminate the influence of confounding factors on serum PGI, PGII, and G-17, we defined the nationality (Han Chinese) and age range (50–60 years). From the serological level, the PGI and PGII levels were significantly higher in the type I *H. pylori* infection group than those in the type II *H. pylori* infection group, which suggested that type I *H. pylori* infection may involve more extensive PGI and PGII cells (gastricum) in the stomach. The PGR level of the type I *H. pylori* infection group was significantly lower than those in the type II *H. pylori* infection group, which suggested that the type I *H. pylori* infection may have a high level of inflammatory activity. There was no significant difference in serum G-17 levels between the type I *H. pylori* infection group and the type II *H. pylori* infection group, which suggested that different types of *H. pylori* infection may have the same range of infection in the antrum. Serological studies suggested that the type I *H. pylori* infection may have a higher ability to cause atrophy and a larger infection range.

**Figure 5:**
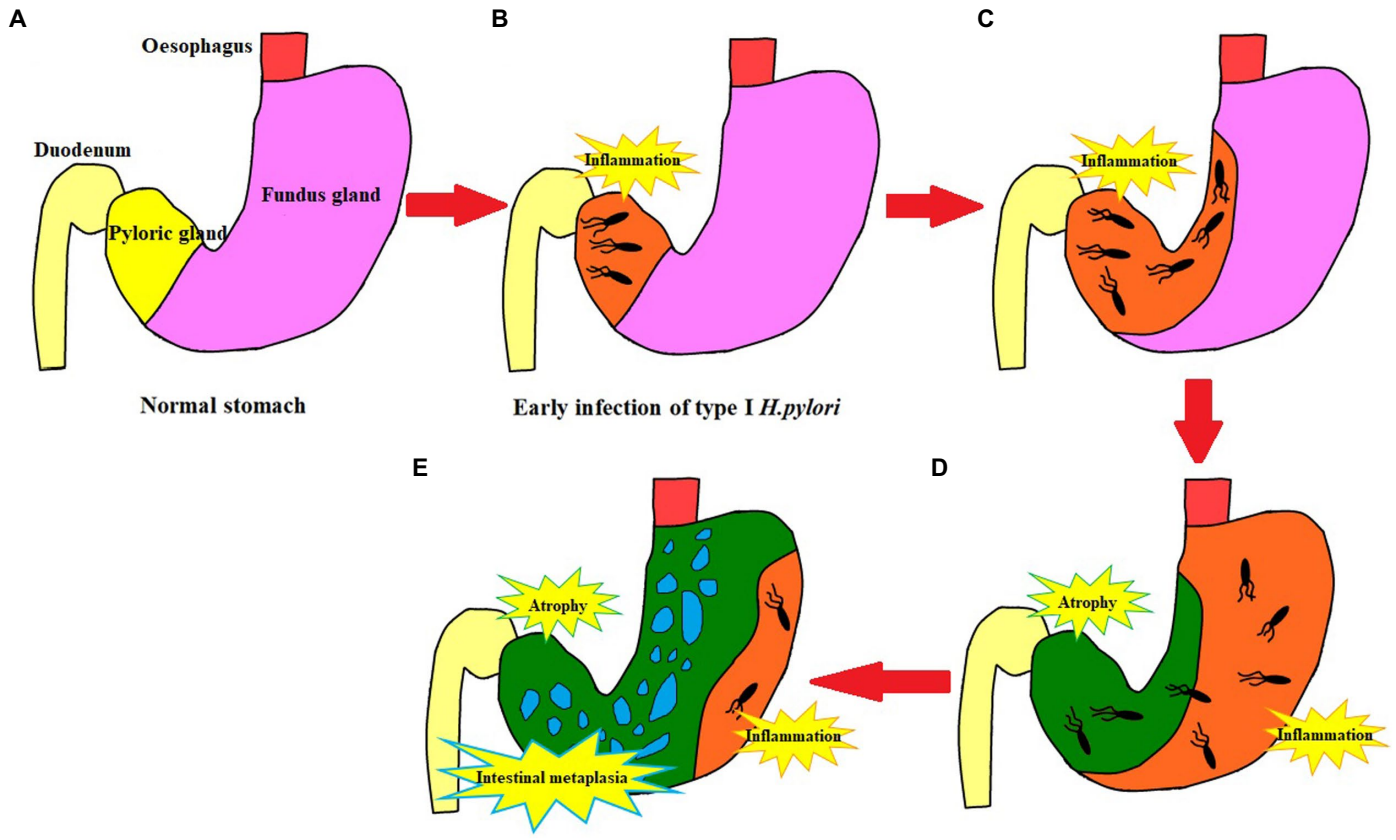
Schematic diagram of type I *H. pylori* infection. **(A)** Normal stomach structure. **(B)** The initial stage of type I *Helicobacter pylori* infection is limited to the gastric antrum. **(C)** Type I *H. pylori* infection progresses to the lesser curvature of the stomach. **(D)** Lesser curvature atrophy; Type I *H. pylori* infection extends from lesser curvature to greater curvature; Inflammation affects the whole stomach. **(E)** With the lesser curvature as the center, the atrophy extends to the periphery, accompanied by intestinal metaplasia and persistent active inflammation in the greater curvature.

**Figure 6:**
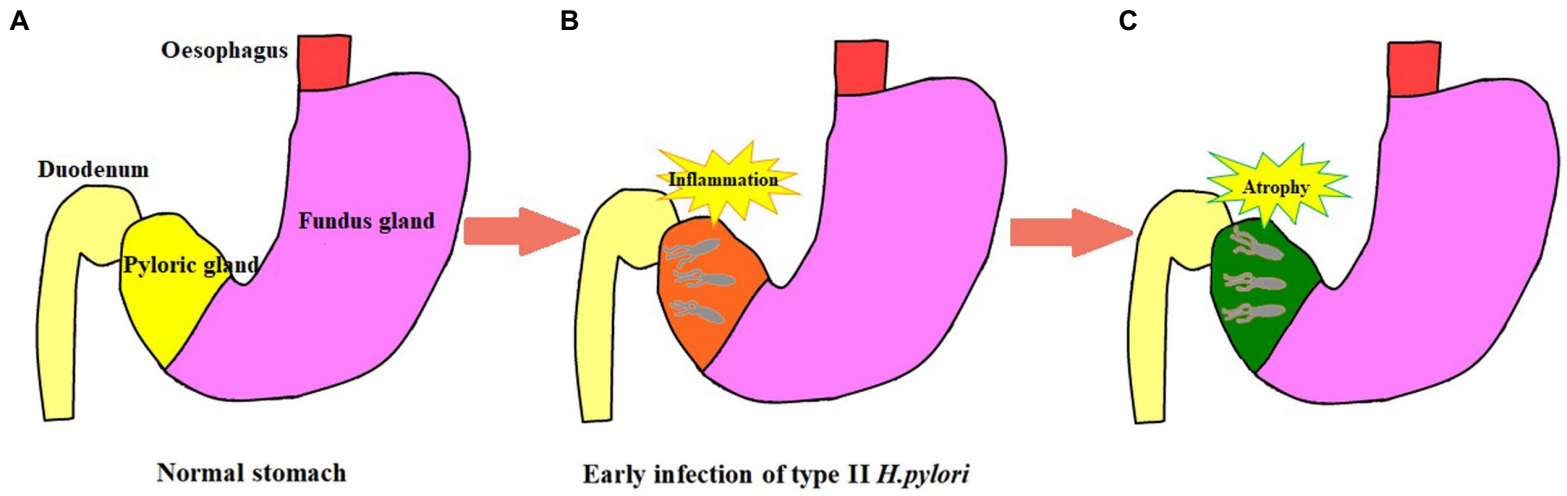
Schematic diagram of type II *H. pylori* infection. **(A)** Normal stomach structure. **(B)** The initial stage of type II *H. pylori* infection is limited to the gastric antrum. **(C)** Type II *H. pylori* infection in gastric antrum progresses to atrophy and it is difficult to advance to the stomach body.

In 2005, the international atrophy research group proposed the OLGA staging system for chronic gastritis; and in 2010, the OLGIM staging system was proposed for intestinal metaplasia to replace atrophy. This is a semi-quantitative scoring method based on the updated Sydney system for chronic gastritis for inflammation and atrophy, which represents the range and degree of gastric mucosal atrophy (Capelle et al., 2010). In this study, to further explore if type I *H. pylori* infection has a higher ability to cause atrophy and a larger infection range or not, different types of *H. pylori* infection were analyzed to determine their ability to cause atrophy and infection range using pathology, non-magnification white light endoscopy combined with Kimura–Takemoto classification, and the absence of RAC (Ebigbo et al., 2021; Xiao et al., 2021). The type I *H. pylori* infection was graded higher than the type II *H. pylori* infection in the Kimura–Takemoto classification, which suggested that the type I *H. pylori* infection may be apt to cause atrophic lesions in the gastricum. Based on the lack of RAC, the type I *H. pylori* infection involved a wider loss of the gastricum than that in the type II *H. pylori* infection, suggesting that the type I *H. pylori* infection had a higher expression level in the gastricum. In the OLGA/OLGIM staging systems, the type I *H. pylori* infection graded higher than the type II *H. pylori* infection in the OLGA staging system, but no significant difference in the OLGIM staging system. The endoscopic and histopathologic data presented earlier provide us with more intuitive evidence. The type I *H. pylori* infection has a higher ability to cause atrophy and a larger infection range than that of the type II *H. pylori* infection, while the type II *H. pylori* infection is limited to the antrum with weaker atrophy progression (as shown in Figures 5, 6).

There are limitations to this study. First, this research did not conduct a prospective study, nor did include different ethnic groups, genders, and ages for follow-up study. Second, the identification of different types of *H. pylori* was determined only by ^14^C-urea Breath Test and *H. pylori* antibody typing classification, lacking gene characteristic information. Third, although the characteristics of *H. pylori* with different virulence from clinical samples are supported by serum gastric function tests, pathological examinations, and endoscope detection, basic experimental verification is still lacking. Based on this, type II *H. pylori* is less toxic, and it causes a slow progression of gastric mucosa atrophy. The support of long-term clinical follow-up data is needed to determine whether asymptomatic patients with type II *H. pylori* infection need eradication therapy or not.

The virulence factors of *H. pylori* not only participate in the induction of inflammatory responses but also control and regulate these responses, maintain chronic inflammation, and most importantly facilitate the interaction among the host, gastric microenvironment, and bacterial virulence factors (Baj et al., 2020). At present, the cancer-promoting mechanism of the CagA protein has been revealed, and only with the earlier eradication of *H. pylori* can we prevent the occurrence of gastric cancer (Takahashi-Kanemitsu et al., 2020). While about 4.4 billion people worldwide have been infected with *H. pylori*, less than 20% of them have developed serious gastric problems and 1% of them have developed gastric cancer (Sharndama and Mba, 2022). Nowadays, numerous *H. pylori-*related guidelines recommend the national eradication of *H. pylori*; yet, there are still many countries that are under great pressure on public health, and eradicating *H. pylori* nationally may pose a potential risk of antibiotic abuse (Kato et al., 2019; Jung et al., 2021; National Clinical Research Center for Digestive Diseases (Shanghai) et al., 2021). Eradicating *H. pylori* infection is still controversial in inflammatory bowel disease, gastroesophageal reflux disease, asthma, and other diseases (He et al., 2022). Moreover, the benefits and risks of eradicating *H. pylori* vary among individuals (Helicobader pylori Study Group, Chinese Society of Gastroenterology, Chinese Medical Association, 2022). In addition, the virulence factor of *H. pylori* plays a key role in its pathogenicity, so individual virulence factor-based eradication therapy may be a better choice in future.

## Data availability statement

The raw data supporting the conclusions of this article will be made available by the authors, without undue reservation.

## Ethics statement

The studies involving human participants were reviewed and approved by Ethics Committee of People’s Hospital of Xinjiang Uygur Autonomous Region. The patients/participants provided their written informed consent to participate in this study.

## Author contributions

WL and WK: conceptualization, data analysis, and manuscript preparation. WH and QJ: data collection. CW, HS, and FG: critical manuscript review. All authors contributed to the article and approved the submitted version.

## Funding

This study was sponsored by the Natural Science Foundation of Xinjiang Uygur Autonomous Region (2019D01C110).

## Conflict of interest

The authors declare that the research was conducted in the absence of any commercial or financial relationships that could be construed as a potential conflict of interest.

## Publisher’s note

All claims expressed in this article are solely those of the authors and do not necessarily represent those of their affiliated organizations, or those of the publisher, the editors and the reviewers. Any product that may be evaluated in this article, or claim that may be made by its manufacturer, is not guaranteed or endorsed by the publisher.
