# Supplementary material for: Characteristics of different types of Helicobacter pylori: New evidence from non-amplified white light endoscopy
Source: Front Microbiol. 2023 Jan 13;13:999564. doi: 10.3389/fmicb.2022.999564 (PMC9881747; doi:10.3389/fmicb.2022.999564)
Supplement: Supplementary file 1 [file Data_Sheet_1.doc]

**
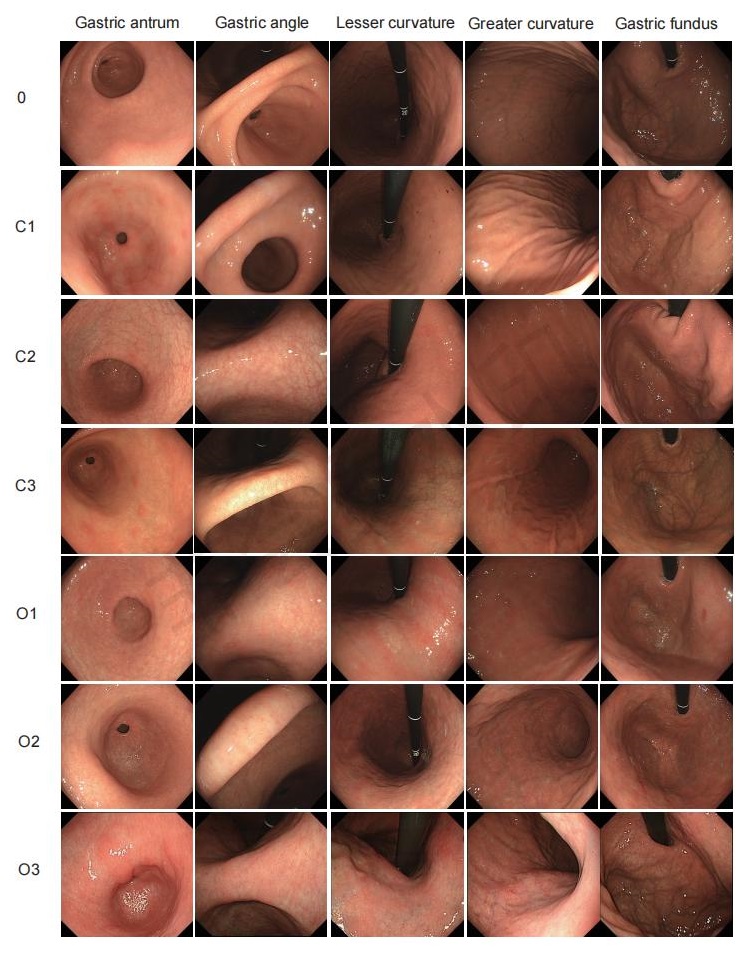
**

**Appendant.1 The range and degree of endoscopic atrophy to the Kimura- Takemoto classification**

**
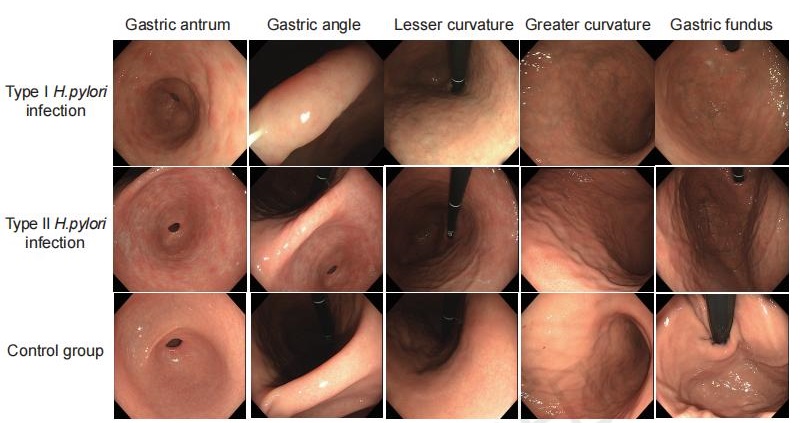
**

**Appendant.2 The status and range of *H. pylori* infection with the characteristics of endoscopic RAC**

**
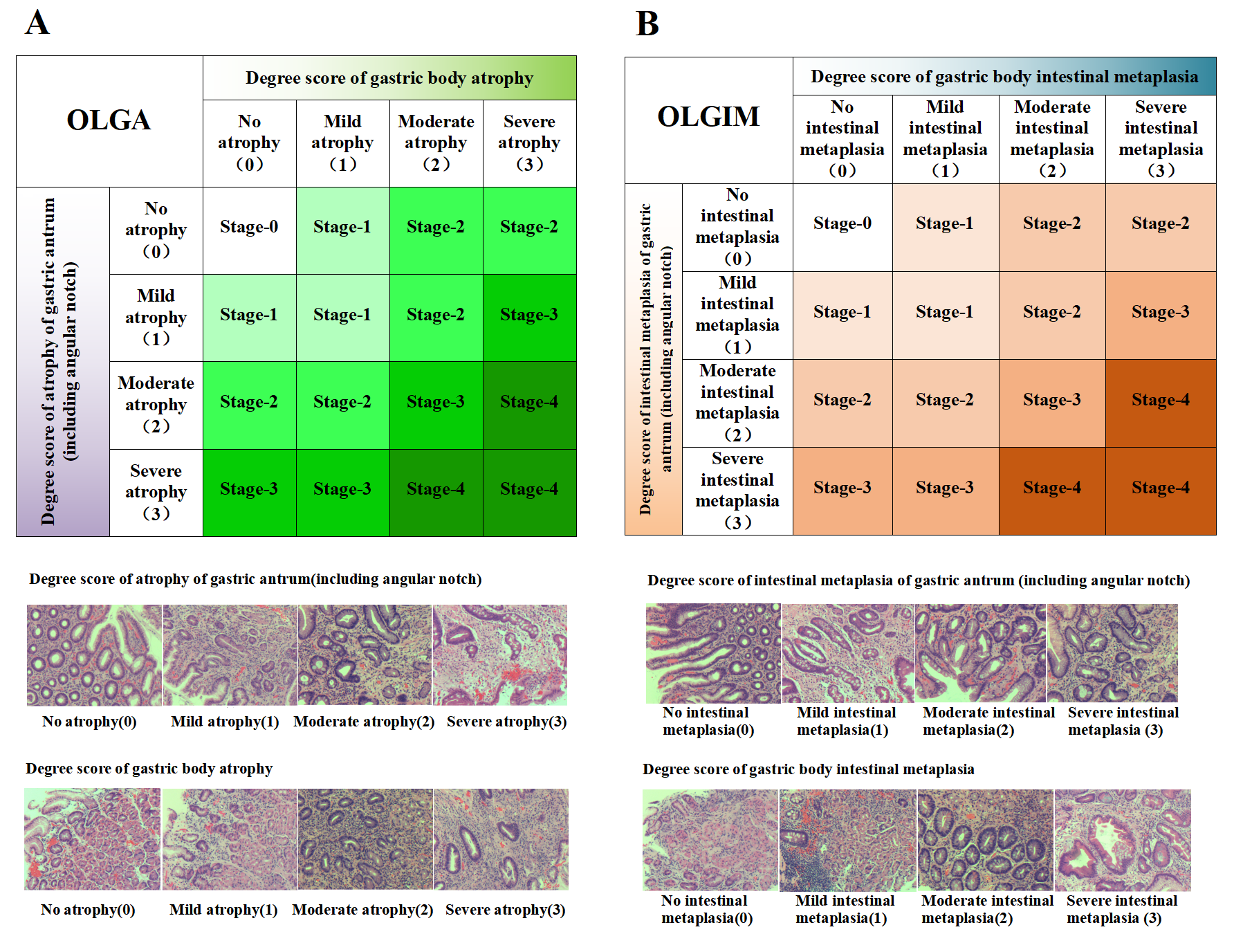
**

**Appendant.3 The OLGA and OLGIM staging systems**
